# Correction: Non-GABA sleep medications, suvorexant as risk factors for falls: Case-control and case-crossover study

**DOI:** 10.1371/journal.pone.0259430

**Published:** 2021-10-27

**Authors:** Yoshiki Ishibashi, Rie Nishitani, Akiyoshi Shimura, Ayano Takeuchi, Mamoru Touko, Takashi Kato, Sahoko Chiba, Keiko Ashidate, Nobuo Ishiwata, Tomoyasu Ichijo, Masataka Sasabe

There are errors in the author affiliations. The correct affiliations are as follows:

Yoshiki Ishibashi^1,2^, Rie Nishitani^1^, Akiyoshi Shimura^3,4^, Ayano Takeuchi^2^, Mamoru Touko^1^, Takashi Kato^5^, Sahoko Chiba^1^, Keiko Ashidate^1^, Nobuo Ishiwata^1^, Tomoyasu Ichijo^1^, Masataka Sasabe^1^

**1** Department of Internal Medicine, Kudanzaka Hospital, Company Overview of Federation of National Public Service Personnel Mutual Aid Associations, 1-6-12, Kudan-minami, Chiyoda-ku, Tokyo, 102–0074 Japan, **2** Department of Preventive Medicine and Public Health, School of Medicine, Keio University, 35 Shinanomachi, Shinjuku-ku, Tokyo, 160–8582 Japan, **3** Department of Psychiatry, Tokyo Medical University, 6-7-1 Nishi-shinjuku, Shinjuku-ku, Tokyo, 160–0023 Japan, **4** Department of Sleep and Psychiatry, Kanno Hospital, 28–1 Honcho, Wako-shi, Saitama, 351–0114 Japan, **5** Department of pharmacy, Kudanzaka Hospital, Company Overview of Federation of National Public Service Personnel Mutual Aid Associations, 1-6-12, Kudan-minami, Chiyoda-ku, Tokyo, 102–0074 Japan.

## References

[1] Ishibashi Y, Nishitani R, Shimura A, Takeuchi A, Touko M, Kato T, et al. (2020) Non-GABA sleep medications, suvorexant as risk factors for falls: Case-control and case-crossover study. PLoS ONE 15(9): e0238723. 10.1371/journal.pone.0238723 32916693 PMC7486134

