# Supplementary material for: Non-GABA sleep medications, suvorexant as risk factors for falls: Case-control and case-crossover study
Source: PLoS One. 2020 Sep 11;15(9):e0238723. doi: 10.1371/journal.pone.0238723 (PMC7486134; doi:10.1371/journal.pone.0238723)
Supplement: S1 Fig — (PPTX) [file pone.0238723.s001.pptx]

## Slide 1
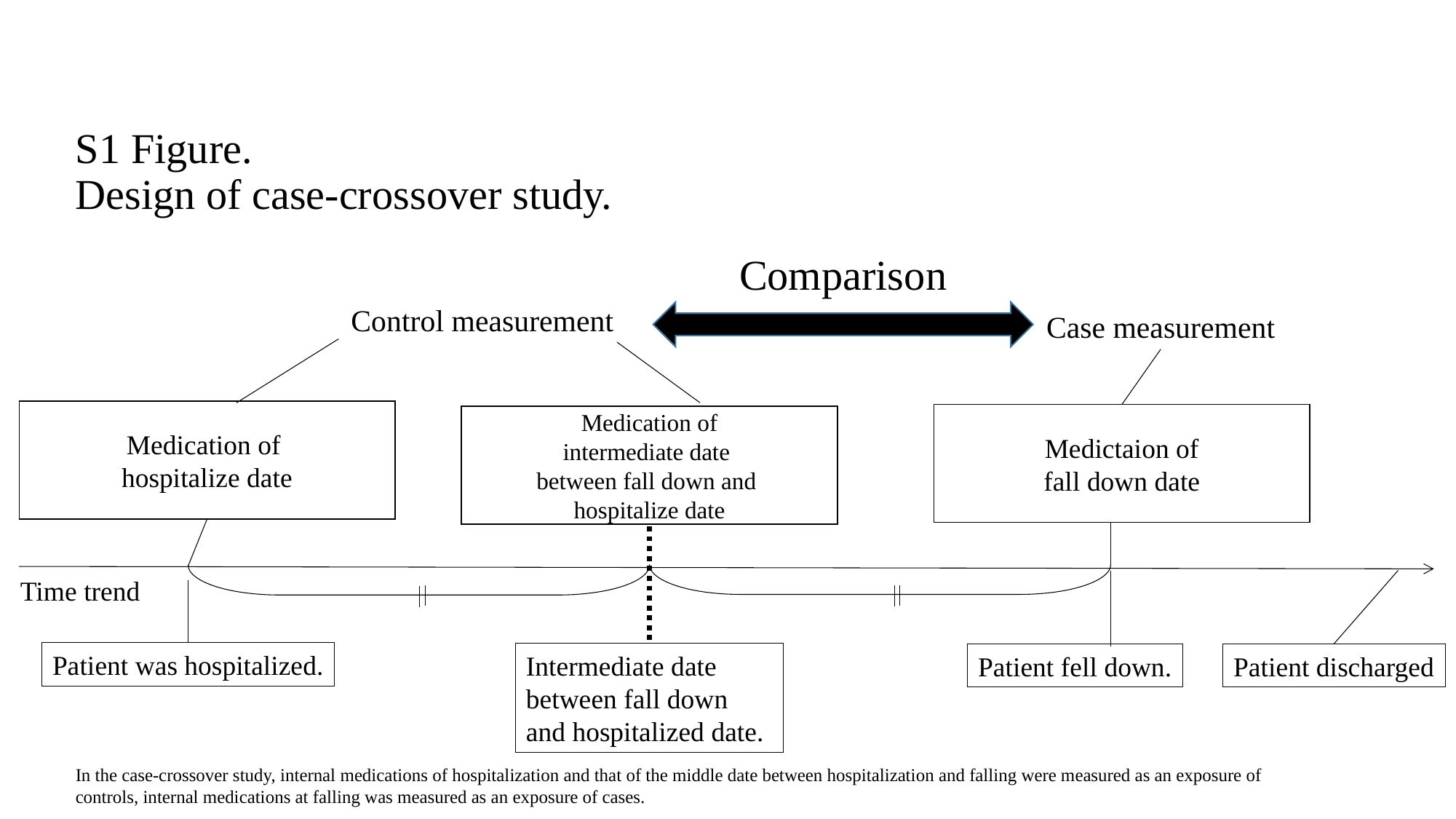

# S1 Figure. Design of case-crossover study.
Comparison
Control measurement
Case measurement
Medication of
hospitalize date
Medictaion of
fall down date
Medication of
intermediate date
between fall down and
hospitalize date
Time trend
Patient was hospitalized.
Intermediate date between fall down and hospitalized date.
Patient fell down.
Patient discharged
In the case-crossover study, internal medications of hospitalization and that of the middle date between hospitalization and falling were measured as an exposure of controls, internal medications at falling was measured as an exposure of cases.
