# Supplementary material for: Non-GABA sleep medications, suvorexant as risk factors for falls: Case-control and case-crossover study
Source: PLoS One. 2020 Sep 11;15(9):e0238723. doi: 10.1371/journal.pone.0238723 (PMC7486134; doi:10.1371/journal.pone.0238723)
Supplement: Attachment — Submitted filename: Comments.docx [file pone.0238723.s011.docx]

The authors analyzed the relationship between non-GABA sleep medications including suvorexant and ramelteon and fall risk in hospitalized patients using logistic regression analysis, and reported that suvorexant and non-BZ sleep medications increased the risk of falls. Furthermore, they verified the certainty of the results using case-cross over analysis and sensitivity analysis.

The study design has been devised to increase the robustness of the results and the sample size is sufficient. The findings of the present study can be an index for prescribing safe sleep medications in actual medical practice. However, I recommend that this paper not be accepted without major revision.

Major comments

(1) The reviewer thinks that the title “Non-GABA sleep medications suvorexant and ramelteon as risk factors for falls in elderly hospitalized patients: case-control and case-crossover study” needs to be reconsidered because it gives the following misunderstandings and false impression.

Ramelteon increased the risk of falls…? (Although ramelteon did not significantly increase the risk of falls in any analyses.)

The study was intended to target the elderly from the beginning…? In that case, it is necessary to describe the range of target age in Methods.

(2) The description of the Methods as a whole takes effort to understand. The reviewer thinks that the authors should separate the sections or paragraphs in a little more detail and clarify what is defined or explained. It may be an option to summarize the difference in the method of surveying items for each target group in a table. Regarding statistical analysis, the reviewer thinks the authors should clarify which of the three statistical analyses is explained.

In Abstract, the description of Methods is also insufficient. The selection criteria of the target, the types of sleep medications investigated, the statistical analysis methods should be described.

(3) In the case-crossover analysis, age, sex and clinical department were adjusted, and 5 types of non-GABA sleep medications were included as independent variables. Since the case-control analysis showed that case-group had significantly higher fall risk scores, the reviewer thinks that the subscores (of 8 items) of the fall risk score (as factors that are likely to contribute to fall risk) should be included in the case-crossover analysis as independent variables.

In addition, the reviewer thinks that the correlations between covariates should also be presented to evaluate confounding and to interpret the whole results.

Minor comments

(1) In Introduction, the authors mentioned attention decline as a cause of falls in the elderly. The reviewer thinks that muscle weakness and sensory deficits are also important factors.

(2) Yokukansan is sometimes written as Yi-gan san. It is better to add it?

(3) The authors mentioned “the number of patients with falls was extremely small. The study which recorded most falls compared 12 and 18 falls” as limitations of previous studies (p. 14, line 16-17). What is the percentage?

(4) The authors hypothesized that falls due to the use of suvorexant might have induced REM sleep behavior disorder and led to falling from beds (p. 15, line 14-17). The reviewer wonders how many, in the authors’ data, fell from beds and what percentage of those were taking suvorexant.

(5) Missing punctuation and capitalization were found. The reviewer also recommend the authors to proofread English by native speakers.
