# Supplementary material for: Non-GABA sleep medications, suvorexant as risk factors for falls: Case-control and case-crossover study
Source: PLoS One. 2020 Sep 11;15(9):e0238723. doi: 10.1371/journal.pone.0238723 (PMC7486134; doi:10.1371/journal.pone.0238723)
Supplement: Attachment — Submitted filename: Response_to_Reviewers_20200819v2.docx [file pone.0238723.s013.docx]

August 19, 2020

Academic editor

Amir Radfar

*PLoS ONE*

Dear Professor, Amir Radfar

Thank you for the opportunity to submit a revised version of our manuscript. Please see below our responses to the specific comments from the reviewers.

Response to Reviewers

**#Reviewer 3**

**Just one trivial point to be re-checked; S1 and S3 Figures are "Table", not "Figure".**

Response: Thank you for your comments. We modified the manuscript, table, and figure as your suggestion.
